# Custom 3D-Printed Cutting Guides for Femoral Osteotomy in Rotational Malalignment Due to Diaphyseal Fractures: Surgical Technique and Case Series

**DOI:** 10.3390/jcm10153366

**Published:** 2021-07-29

**Authors:** Jaime Oraa, Maider Beitia, Nicolás Fiz, Sergio González, Xabier Sánchez, Diego Delgado, Mikel Sánchez

**Affiliations:** 1 Arthroscopic Surgery Unit, Hospital Vithas Vitoria, 01008 Vitoria-Gasteiz, Spain; jaime.oraa@ucatrauma.com (J.O.); nicolas.fiz@ucatrauma.com (N.F.); sergio.gonzalez@ucatrauma.com (S.G.); 2 Advanced Biological Therapy Unit, Hospital Vithas Vitoria, 01008 Vitoria-Gasteiz, Spain; maider.beitia@ucatrauma.com (M.B.); diego.delgado@ucatrauma.com (D.D.); 3 Osteomodel, 20018 San Sebastian, Spain; xabier.sanchez@osteomodel.com

**Keywords:** 3D printing, 3D technology, femoral osteotomy, femoral malrotation, femoral anteversion, femoral shaft fractures

## Abstract

Femoral shaft fractures are one of the most common injuries in trauma patients. The gold standard treatment consists of closed reduction and intramedullary nailing, providing a high fracture healing rate and allowing early mobilization. However, rotational malalignment is a well-known complication following this procedure, and excessive femoral anteversion or femoral retroversion can trigger functional complaints. In order to achieve the ideal degree of femoral rotation, a 3D planning and printing cutting guides procedure was developed to correct femoral malrotation. A patient series with malalignment after a femoral diaphyseal fracture was operated on with the customized guides and evaluated in this study. Computed tomography scans were performed to accurately determine the number of degrees of malrotation, allowing the design of specific and personalized surgical guides to correct these accurately. Once designed, they were produced by 3D printing. After surgery with the customized guides to correct femoral malrotation, all patients presented a normalized anteversion angle of the femur (average −10.3°, range from −5° to −15°), according to their contralateral limb. These data suggest that the use of customized cutting guides for femoral osteotomy is a safe and reproducible surgical technique that offers precise results when correcting femoral malrotation.

## 1. Introduction

Femoral shaft fractures (FSF) are one of the most common injuries in trauma patients, with an incidence of between 10 and 21 per 100,000 people per year [1,2]. Their causes are often related to high energy mechanisms such as traffic accidents and are commonly associated with multiple injuries, life-threatening complications, sequelae and limb deformities, namely shortening and malrotation, if not treated appropriately [3].

The gold standard treatment for FSF consists of closed reduction and intramedullary nailing. This technique provides a high fracture healing rate and allows early mobilization [4,5]. However, a rotational malalignment is a well-known complication following this procedure, and a difference in rotation greater than 15° compared with the healthy side can be responsible for functional complaints [6,7]. This complication may occur in 28% of the patients [8], although other studies showed that the incidence of malrotation after intramedullary nailing for femur fractures ranges from 19% to 56% [9,10,11]. Femoral malrotation is calculated by measuring the femoral version, which is defined according to the technique described by Jeanmart et al., determining the angle between a line tangential to the dorsal bony contours of the femoral condyles and a line drawn through the axis of the femoral neck [12,13].

The average values of femoral anteversion range from 10° to 15°, and exceeding these values on both sides can lead to pathological conditions [14]. On the one hand, excessive femoral anteversion can imply anterior knee pain and patellofemoral instability, anterior hip pain and labral tears in patients with concomitant femoro-acetabular impingement, posterior extra-articular hip impingement and ischiofemoral impingement. It is also a frequent reason for an internally rotated gait, which can cause discomfort when walking, with tripping, and difficulties with running and doing sports. On the other hand, a lack of femoral anteversion (or femoral retroversion) can cause damage to the labrum and articular cartilage of the hip and early osteoarthritis, and an externally rotated gait [7,15,16,17,18,19,20,21,22,23,24,25,26].

By means of derotation osteotomies, surgeons seek to resolve the malrotation resulting from the initial surgery for FSF. However, achieving the ideal degree of femoral version is difficult and challenging. One of the reasons is that current surgical techniques for correcting malrotation are observer-dependent, based on measurements of intraoperative clinical and radiological parameters [27,28,29,30,31].

Therefore, new techniques need to be developed to allow for more accurate correction. In this regard, the use of custom 3D planning and printing cutting guides is a novel tool in surgical interventions to correct femoral malrotation. Advances in 3D technology in recent years have led to an exponential increase in its use in medicine, and especially in orthopedic surgery [32,33,34,35].

3D printing is an additive manufacturing technique that allows us to transform a digital model into a three-dimensional object. Three-dimensional models are obtained by processing digital radiological studies of patients, such as computed tomography (CT) scans, and when the virtual model has been obtained, it can be printed. Objects are built layer by layer, using different technologies and materials depending on the final application for which they are intended. 3D printing allows manufacturing by successively adding material to the object, so as to create complex structures that could not be obtained with other technologies [36].

CT scans can accurately determine the degree of malrotation, enabling the design of a specific and personalized surgical guide. Its design and 3D printing according to the surgical plan would improve the predictability of osteotomy procedures [37,38]. Another option to solve this problem could be the use of navigated surgery, but, to our knowledge, there are so far no publications on this technique.

In the present study, we describe in detail a new surgical technique based on the design and 3D printing of customized cutting guides for femoral osteotomies with rotational malalignment after a diaphyseal fracture, and the clinical outcomes in a case series.

## 2. Materials and Methods

### 2.1. Patients

Six patients with a medical history of closed FSF after a traffic accident are described in Table 1. Five of them had undergone surgery at other centers, and were brought into our clinic for a second opinion; the other patient did not have any previous surgery.

Clinical examination showed groin and/or knee pain, with an in-toe or out-toe gait. On the initial telemetry all the patients presented signs of femoral malrotation, so a CT scan was performed to calculate the degree of femoral torsion.

### 2.2. Design and 3D Printing

**Figure 1:**
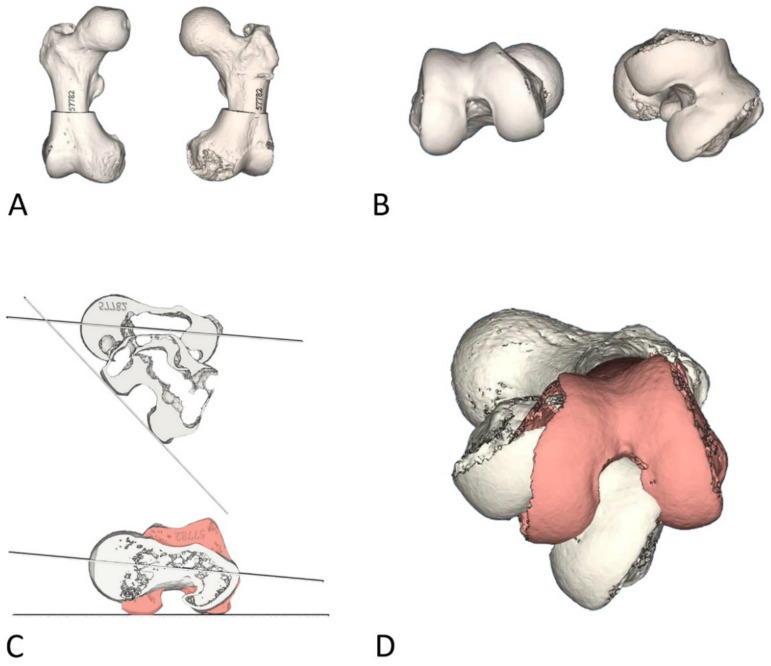
Calculation of correction degrees (Patient 3). A 3D composition of the femoral head, proximal metaphysis and condyles of both lower extremities are obtained from CT images (**A**), and the rotational malalignment of both limbs is evaluated (**B**). The degrees of malrotation are calculated by software according to Jeanmart’s technique (top, (**C**)), as well as the degrees needed for correction (red) (bottom, (**C**)). Once the position of the distal femur is established before and after (red) rotation correction (**D**), the guides are designed to correct the necessary number of degrees and to fit the patient’s bone surface.

The CT images were treated with 3D reconstruction software (Mimics^®^, Materialise, Belgium), obtaining a 3D composition of the femoral head, proximal metaphysis and condyles of both lower extremities (Figure 1A). The rotational malalignment was accurately measured by the software according to Jeanmart’s technique, as described above [12], and compared with the contralateral limb (Figure 1B).

After quantifying the required degree of correction, preoperative planning was performed (Figure 1C,D). A diaphyseal derotational osteotomy with intramedullary fixation was considered for all the patients. A segmentation process was performed, in which 3D volume is generated from a CT scan, and custom surgical guides were made for the correction of the femoral rotation.

The planning, design and manufacturing process is divided into different phases. Initially, the desired anatomical area, in this case the femur, is segmented. The Mimics Innovation Suite from Materialise is used for this purpose. Subsequently, using Nx Unigraphics from Siemens and Magics (Materialise, Materialise, Belgium; NX Unigraphics, Munich, Germany), the surgery is planned and simulated. During this phase we compare different strategies and results in order to obtain an optimal outcome.

Once the correction is defined, customized guides are designed with Nx Unigraphics and Magics. Depending on the bone deformity, these can be placed separated or joined together as required to facilitate placement during the surgical procedure. After defining the design, all components are manufactured in biocompatible ABS M30i with a Stratasys F380mc printer and after undergoing a validated cleaning process, they are sent to the hospital for sterilization by a Low Temperature Hydrogen Peroxide and Plasma sterilizer (Matachana 130 HPO^®^) and subsequent surgical use.

### 2.3. Surgical Technique

**Figure 2:**
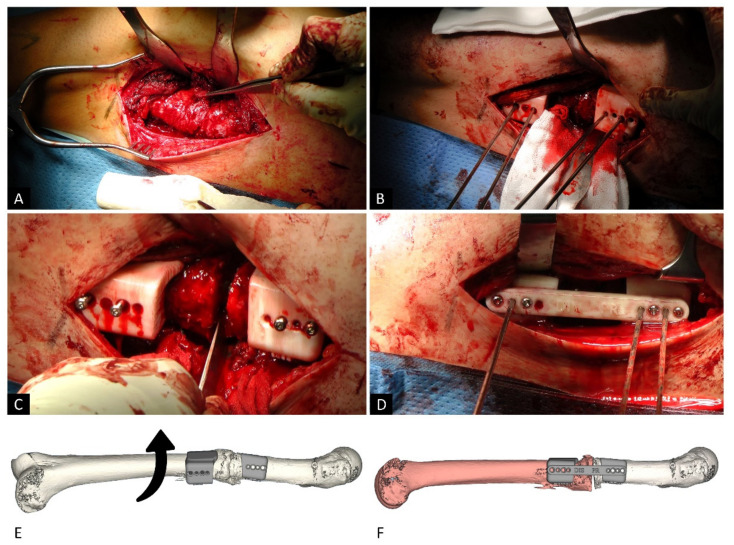
Surgical technique (Patient 3). After exposing the femoral diaphysis through a postero-lateral approach (**A**), the two initial surgical guides are pinned to the bone surface (**B**). Subsequently, the osteotomy is carried out by removing all previous osteosynthesis material (**C**). The correction is performed with an external or internal rotation of the distal femoral fragment and the third 3D- printed guide is used to connect the other two, providing the correct femoral rotation degree (**D**). The degrees of rotation of the distal femoral fragment are defined by the alignment of the two guides (**E**). Once the two guides are aligned and connected by the third piece, the distal fragment is already correctly rotated according to the surgical planning (**F**). Finally, the osteotomy is completed by inserting the intramedullary nail and removing the surgical guides.

The femoral diaphysis was exposed through a postero-lateral approach, between the vastus lateralis and lateral intermuscular septum (Figure 2A). The two initial surgical guides were pinned to the bone surface with two monocortical Kisrchner wires and two monocortical screws for each piece (Figure 2B). The 3D-printed guides adapted accurately to osteophytes and fracture lips, ensuring perfect rotational positioning and precision in placement.

The next step was to remove previous osteosynthesis material (T2 femoral nail, Kuntscher nail or Russell-Taylor nail). After conducting a femur osteotomy through the previous fracture site, correction of internal or external femoral torsion was performed with an external or internal rotation of the distal femoral fragment, respectively (Figure 2C–E). Then, the third 3D-printed guide was used to connect the other two, and provide the correct femoral rotation degree (Figure 2D–F). Finally, a new T2 nail (Stryker) was introduced, with both proximal and distal locking. As the screws in the guides were monocortical, it was not necessary to remove the guides to place the new nail, which maintained the correction and provided stability for the derotation femoral osteotomy. Once the nail was placed, the surgical guides were removed, and correct positioning was checked under fluoroscopy.

**Figure 3:**
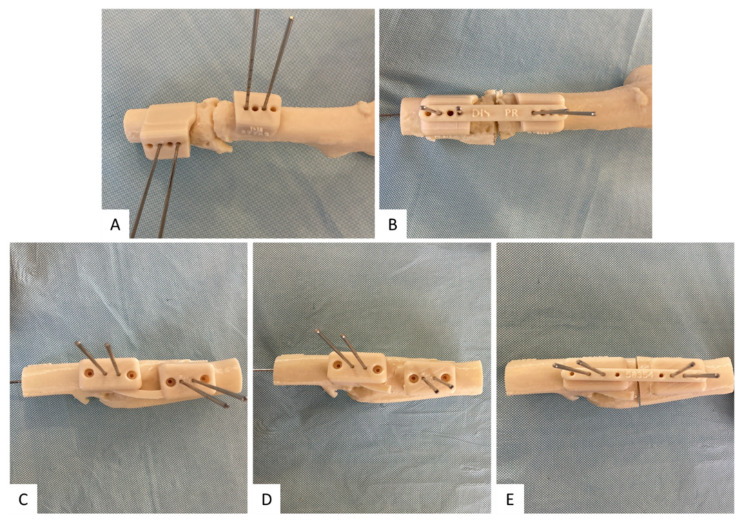
Surgical procedure. Guides are fixed separately in cases of fracture sequelae to correctly adapt them to the relief of the bone (**A**) and then the rotation is performed until the wires are aligned (**B**). In cases of idiopathic anteversion without fracture and in cases with poorly exuberant callus bone, the guide is first fixed with the pieces joined together (**C**), and then separated (**D**) and rotated until the alignment of the wires is achieved (**E**).

In cases of fracture sequelae, it may be easier to apply the guides separately to better adapt each part of the guide to the deformed relief of the bone. Furthermore, in cases of idiopathic anteversion without fracture and in cases with poorly exuberant callus bone, the different parts of the guide are first joined together and then separated before correcting the rotation (Figure 3).

**Figure 4:**
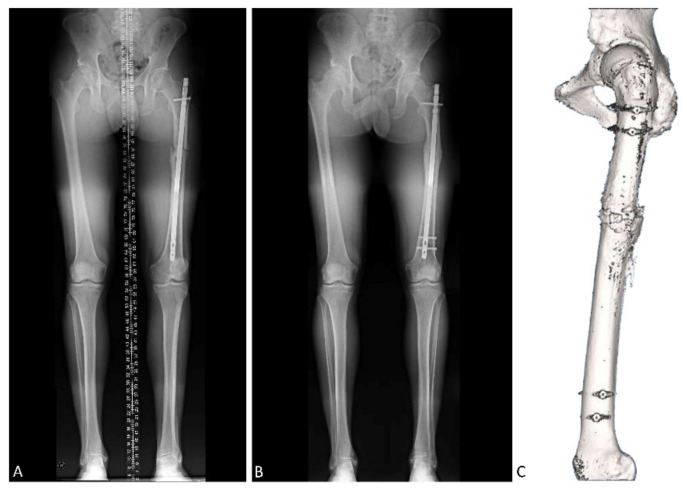
Pre- and postoperative image study (Patient 3). Teleradiographs are conducted to observe rotation before (**A**) and after surgery (**B**). It also indicates callus formation over time. The CT scan also indicates whether the alignment of the femur is correct after surgery (**C**).

The success of the surgery is assessed both clinically and radiologically. Thus, X-rays are employed to see the consolidation status of the osteotomy and, additionally, teleradiography is performed to confirm the rotation. This rotation is then compared with the initial one. As for the physical examination, the hip is explored for internal and external rotation and to assess the symmetry between both hips (Figure 4).

## 3. Results

Results are shown in Table 1. A total of six derotation osteotomies were performed in six patients: two female and four male. The average age was 43 years (range 23–72 years). Three of the patients presented a femoral external rotation deformity (average +28°, range from +1° to +43°), while the other three patients displayed an internal rotation deformity of the femur (average −43°, range from −24° to −60°). After surgery, all patients presented a normalized anteversion angle of the femur (average −10.3°, range from −5° to −15°), with respect to their contralateral limb.

As explained before, the etiology was post-traumatic in all the patients, in each case due to a traffic accident, and five of the six patients had previously undergone surgery. The mean time elapsed from the first surgery to the surgery performed by our medical team was 179.2 months (range 10–600 months). Three of the patients had a T2 nail (Stryker) implanted at the first operation, another patient had a Russell-Taylor nail (Smith & Nephew), while the remaining patient had a Küntscher nail. No major complications had occurred in this time (after first surgery), nor any deep or superficial infections.

## 4. Discussion

This study describes a new surgical technique to correct femoral malrotation using custom 3D-printed cutting guides. In addition, we present a series of six patients who consulted for femoral malalignment after an FSF, in which a femoral derotation osteotomy was performed using the customized guides. The results suggest this is a safe process with great precision to establish the proper rotation of the femur. Using 3D-printed guides makes the surgery shorter and technically easier, with less radiation inside the operating room. Furthermore, this procedure is inexpensive.

Customized osteotomy guides solve one of the major difficulties when correcting malrotations by providing a highly accurate calculation and correction of the degrees of malrotation. Several methods of calculating intraoperative femoral rotation were published in the literature. A work by Krettek et al. described simple and useful techniques used to analyze limb alignment after initial fixation of femoral and tibial fractures [39]. Jagernauth et al. used a protractor to correct the femoral internal rotation after intramedullary nailing, performing a derotation osteotomy leaving the previous nail in situ [40]. A method carried out by Espinoza et al. set femoral rotation in acute fractures using the inherent anteversion of the intramedullary nail [41]. Stambough et al. determined femoral anteversion measuring the trochanteric prominence angle in adolescents with symptomatic excessive femoral anteversion [42]. Although all these techniques offer a variety of possibilities for solving the proposed challenge, they present some limitations, such as the requirement of a high dose of radiation in the operating room, or the help of an experienced radiology technician to achieve the correct visualization of the necessary projections. However, the major drawback of these techniques is that the correction to be performed is observer-dependent and, therefore, it should be taken into consideration that these techniques are somewhat susceptible to error.

On the other hand, the technique described in the present study calculates the degrees to be corrected by means of virtual planning prior to surgery based on the patient’s imaging studies, from which the guides are designed to accurately correct the degrees of rotation as well as fitting the patient’s bone tissue with exactitude. Consequently, the surgeon only needs to follow the indications provided by the custom 3D-printed guide, thus avoiding a subjective estimation of the number of degrees to be corrected during the surgical intervention, and the resulting error. This was confirmed by the fact that patients who underwent this surgical technique achieved a normalized anteversion within the recommended range (from −5° to −15°).

The major disadvantage of this procedure is that it requires open surgery. As a consequence, there is an increased risk of blood loss during the operation as well as a possible risk of delay in the union or nonunion at the level of the osteotomy. This was resolved by Buly et al. by performing 55 femoral derotation osteotomies in 43 patients for version abnormalities using an intramedullary hand saw, with the advantage of not requiring exposure of the osteotomy site [43]. Rotational control was achieved by placing 1/8-inch smooth Steinmann pins into the femur, proximal and distal to the osteotomy to the desired amount of rotational correction. However, the angular correction was again observer-dependent, controlled visually using flat, triangular guides from a blade plate instrument set. Other authors also carried out rotational femoral osteotomies using an intramedullary saw [44,45].

It should be noted that the positioning of the 3D-printed guides is a critical step in this type of surgery [46]. Thus, the detailed and meticulous fabrication of the guides is mandatory, as well as correct positioning in the bone, taking into account the fracture ends and reference bone reliefs used in the design of the guides. Suboptimal intraoperative guide positioning could lead to incomplete or excessive correction.

In the case of bilateral femur malrotation due to bilateral FSF, it is not possible to compare with a healthy limb, and the desired femoral anteversion should be estimated based on the mean values of the population.

In recent years, the techniques of designing and manufacturing surgical guides have been improved, as well as the printing material. This is a modern and personalized technique, in constant evolution, in which different custom guides are manufactured for each case. Its versatility makes it a promising alternative for other types of surgical intervention. It might be a valid surgical treatment of acute fractures with severe comminution of the fracture site, since only a CT scan of both lower extremities is needed, and the preparation time of the guides is relatively short; around 5–6 h. It may even be an option for treatment with osteosynthesis plates or external fixation, when needed, in other pathologies such as hypophosphatemic osteomalacia, osteogenesis imperfecta, polyostotic fibrous dysplasia, vitamin D-resistant hypophosphatemic rickets and other lower extremity bone deformities.

This study has several limitations, including the small sample size, and further studies are needed to evaluate the technique more in greater depth. In addition, it presents the usual shortcomings associated with retrospective studies of this kind.

## 5. Conclusions

The design and 3D printing of customized cutting guides for femoral osteotomies with rotational malalignment after a diaphyseal fracture is a reproducible surgical technique that offers precise results when correcting femoral malrotation. Following surgery, all patients presented a normalized anteversion angle of the femur. The use of patient-tailored surgical guides could be implemented in different types of surgical interventions, improving aspects such as accuracy and surgery times.

## Figures and Tables

**Table 1:** Clinical and surgical data of the patients.

	Patient 1	Patient 2	Patient 3	Patient 4	Patient 5	Patient 6
Age (years)	23	72	40	30	59	35
Sex	Female	Male	Male	Male	Female	Male
Side	Left	Left	Left	Right	Right	Left
Previous distal femur deformity	Internal rotation	External rotation	External rotation	Internal rotation	External rotation	Internal rotation
Pre-angulation (°)	−60	40	43	−44	1	−24
Correction (°)	45	50	50	33	15	19
Post-angulation (ª)	−15	−10	−7	−11	−14	−5

## Data Availability

The data presented in this study are available within the article.
